# The FAT10- and ubiquitin-dependent degradation machineries exhibit common and distinct requirements for MHC class I antigen presentation

**DOI:** 10.1007/s00018-012-0933-5

**Published:** 2012-02-19

**Authors:** Frédéric Ebstein, Andrea Lehmann, Peter-Michael Kloetzel

**Affiliations:** Institut für Biochemie, Charité-Universitätsmedizin Berlin, Campus CVK, Oudenaderstr.16, 13347 Berlin, Germany

**Keywords:** FAT10, Ubiquitin, Immunoproteasomes, PA28, NUB1, Antigen presentation

## Abstract

Like ubiquitin (Ub), the ubiquitin-like protein FAT10 can serve as a signal for proteasome-dependent protein degradation. Here, we investigated the contribution of FAT10 substrate modification to MHC class I antigen presentation. We show that N-terminal modification of the human cytomegalovirus-derived pp65 antigen to FAT10 facilitates direct presentation and dendritic cell-mediated cross-presentation of the HLA-A2 restricted pp65_495–503_ epitope. Interestingly, our data indicate that the pp65 presentation initiated by either FAT10 or Ub partially relied on the 19S proteasome subunit Rpn10 (S5a). However, FAT10 distinguished itself from Ub in that it promoted a pp65 response which was not influenced by immunoproteasomes or PA28. Further divergence occurred at the level of Ub-binding proteins with NUB1 supporting the pp65 presentation arising from FAT10, while it exerted no effect on that initiated by Ub. Collectively, our data establish FAT10 modification as a distinct and alternative signal for facilitated MHC class I antigen presentation.

**Electronic supplementary material:**

The online version of this article (doi:10.1007/s00018-012-0933-5) contains supplementary material, which is available to authorized users.

## Introduction

The production of minimal CD8+ T cell antigenic peptides mostly depends on the degradation of target proteins by the ubiquitin–proteasome system (UPS). In this pathway, covalently attached ubiquitin (Ub) typically marks a substrate protein for degradation by the 26S proteasome, and it has been shown that increased susceptibility to ubiquitylation can facilitate MHC class I antigen presentation in vivo [1–3]. Conjugation of Ub to lysine (K) side chains of target proteins uses the concerted actions of a succession of specific enzymes (E1, E2 and E3) that sequentially transfer the activated Ub to a protein substrate. K48-linked chains are the most abundant forms of poly-Ub chains within the cells and target substrates for 26S proteasome-mediated degradation. The 26S proteasome complex consists of two sub-complexes: the 19S regulatory particle and the 20S particle containing the three catalytic subunits β_1_, β_2_ and β_5_. In mammalian cells, upon induction by type I and/or II interferon (IFN), these constitutive catalytic subunits are replaced by the inducible subunits iβ_1_/LMP2, iβ_2_/MECL1 and iβ_5_/LMP7, forming the immunoproteasome (i-proteasome) [4–6]. Studies of i-proteasome function have revealed that, in most instances, it generates MHC class I-binding peptides more efficiently than standard proteasomes (s-proteasomes) [7–9]. IFN-γ stimulation is also accompanied by increased expression of the proteasome activator PA28, which associates with the 20S proteasome, thereby forming so-called hybrid proteasomes complexes (i.e. 19S-20S-PA28) that enhance the production of antigenic peptides [10, 11].

Over the past decade, a growing number of Ub-like proteins (UBL) sharing structural homology with Ub have been identified, such as NEDD8, SUMO, ISG15 and FAT10 [12, 13]. Like Ub, they exhibit the capacity to be conjugated to K residues in a substrate protein, and are involved in the regulation of diverse cellular processes, including nuclear transport, transcription, stress response, and DNA damage. FAT10 (HLA-F-adjacent transcript 10) is the most recently identified member of the UBL family and, up to now, very little is known about its biological function. FAT10 gene expression has been reported to be under the influence of cytokines, including TNF-α and IFN-γ, and is timely induced during the later phase of dendritic cell (DC) maturation and during apoptosis [14–16]. Increased levels of both free FAT10 and FAT10-protein conjugates have also been reported in various tumours including hepatocellular carcinoma as well as gastric and gynaecological cancers [17, 18]. Although the lymphocytes from FAT10^−/−^ mice were, on average, more prone to spontaneous apoptotic death, no histological differences were found between wild-type and FAT10^−/−^ mice [19]. Within cells, FAT10 is covalently conjugated to cellular proteins in a pathway involving an E1-E2-E3 enzyme cascade, which is only partially characterised. Recently, UBE1L2 and UBE2Z have been reported to function as E1 and E2 enzymes for FAT10, respectively [20, 21], whereas no FAT10-specific E3 enzymes have so far been experimentally verified.

Interestingly, FAT10 shares with Ub the unique ability of targeting substrates for proteasomal degradation [15, 22]. However, there exists no information whether FAT10 modification of a substrate protein may contribute to the peptide supply for MHC class I-restricted antigen presentation. Here, we show that an N-terminal fusion of the human cytomegalovirus (HCMV)-derived pp65 antigen with FAT10 accelerates the proteasomal degradation of pp65 and results in improved presentation of the HLA-A2-restricted pp65_495–503_ epitope. Importantly, the antigen processing pathway used by this FAT10-pp65 fusion protein differs considerably from that used by an Ub-pp65 chimera in terms of i-proteasomes, PA28 and Ub-binding proteins. In summary, our data underscore the importance of the FAT10 conjugation system as an alternative and distinct pathway for MHC class I antigen processing.

## Materials and methods

### Reagents and antibodies

Anti-pp65 (CH12), anti-FAT10 (FL-165) and anti-β-actin (C4) antibodies were purchased from Santa Cruz Biotechnology. Monoclonal anti-LMP2 (LMP2-13), anti-Ub (FK2), anti-Rpn10 (S5a-18) and polyclonal anti-NUB1 antibodies were obtained from Biomol. The polyclonal anti-PA28-β was purchased from Cell Signaling. Anti-HA monoclonal antibody (16B12) was obtained from Covance. Antibodies against PA28-α, MECL1 (K65/4) and LMP7 (K63/5) were from the laboratory stock and used as previously described [23]. MG132 (benzyloxycarbonyl-Leu-Leu-Leu-CHO), *N*-ethylmaleimide (NEM) and phytohaemagglutinin (PHA-L) were all purchased from Sigma. Lipopolysaccharide (LPS) was obtained from Invivogen. Unless specified, all recombinant cytokines used in this study (IL-2, TNF-α, IFN-γ) were purchased from Miltenyi Biotec. The peptide pp65_495–503_ (NLVPMVATV) was custom-synthesised by our peptide synthesis facility (Institute of Biochemistry, Charité, Berlin).

### Cell culture

The stable cell line HeLa A2+ (clone 33) was established in our laboratory and cultivated in Iscove Medium supplemented with 10% FCS in the presence of 2 μg/ml puromycin. The expression of HLA-A2 molecules on the cell surface was determined using flow cytometry, using the BB7.2 mAb (kindly provided by Dr. A. Paschen, Essen, Germany). The clone 33/2 (HeLa A2+/IP) is a derivative of the clone 33 that stably expresses the three inducible subunits LMP2, MECL1 and MECL1 and was maintained in the presence of 2 μg/ml puromycin and 300 μg/ml hygromycin. HEK293 cells were grown in DMEM medium (Biochrom, Berlin, Germany) containing 10% FCS, 2 mM l-glutamine and 100 U/ml penicillin and streptomycin (purchased from PAA Laboratories), as previously described [24]. DC were generated from enriched CD14+ monocytes cultured in the presence of GM-CSF (500 U/ml) and IL-4 (100 U/ml) for 5 days. The pp65 CTL clone 61, specific for pp65_495–509_, was generated from sensitisations of naïve CD8+ T cells with peptide-pulsed DC, as previously described [14]. It was regularly expanded at 37°C (5% CO2) in RPMI 1640 medium supplemented with 8% human serum (Promocell) and recombinant IL-2 (150 U/ml) in the presence of irradiated BLCL cells and allogeneic peripheral blood mononuclear cells (PBMC) and PHA-L (1 μg/ml).

### Plasmids

The full-length sequence of the HCMV-derived pp65 was PCR amplified from the pcDNA6-pp65.35 plasmid (kind gift of B. Plachter, Johannes Gutenberg-University, Mainz, Germany) and cloned into the eukaryotic expression vector pcDNA3.1/*myc*-HIS (version B). To generate Ub-pp65 and FAT10-pp65 fusion proteins, the sequences encoding Ub and FAT10 were PCR amplified from LPS-treated DC cDNA and cloned in frame into the pcDNA3.1/pp65-*myc*-HIS construct. A DNA fragment corresponding to the FAT10 coding sequence was amplified from LPS-treated DC cDNA by PCR using a forward primer encoding the FLAG tag sequence. The PCR-amplified DNA was then cloned into the pcDNA3.1/Zeo(+) expression vector (Invitrogen) to generate a N-terminal FLAG-tagged version of the FAT10 protein. Likewise, the sequence encoding the amino acids 1–76 of human Ub was amplified by PCR using forward primer encoding the epitope tag derived from the influenza HA protein (YPYDVPDY) and cloned into the pEGFPN3 plasmid (BD Clontech), so that a HA-Ub-GFP fusion product can be synthesised following transfection of mammalian cells. The full-length cDNA for NUB1 and NUB1L was amplified by PCR from LPS-treated DC using specific primers containing sequences derived from the 5′ and 3′ portions, including their stop codons and restriction enzyme sites compatible with cloning into pcDNA3.1/myc-HIS expression vector.

### In vitro transfection and western blotting

HeLa, HeLa A2+, HeLa A2+/IP and HEK293 cells were transfected with 4 μg of each plasmid using Lipofectamine 2000 (Invitrogen). Sixteen hours after transfection, cells were rapidly washed in ice-cold PBS and solubilised with a NP40-based lysis buffer (50 mM Tris, 50 mM NaCl, 5 mM MgCl_2_, 100 mM NEM, 10 μM MG132 and 0.1% NP40) for 15 min on ice. The cell lysates were clarified by centrifugation (14,000*g* for 15 min). Protein concentration of supernatants was determined using a BCA™ protein assay kit (Thermo Scientific), and 30 μg proteins were resolved on SDS-PAGE and transfer to PVDF membranes (MilliQ). Membranes were blocked for 30 min in PBS containing 5% milk followed by overnight incubation with primary antibodies. After subsequent washings and incubation with horseradish peroxidase-coupled secondary antibodies, immunoblots were developed with the use of enhanced chemoluminescence (ECL) (Amersham).

### Immunoprecipitation

HeLa cells were transfected with an expression vector encoding a HA-tagged Ub-GFP fusion protein (HA-Ub-GFP) alone or in combination with plasmids encoding *myc*-tagged versions of the pp65, Ub-pp65 or FAT10-pp65 constructs. After a 16-h transfection, whole-cell lysates were made in lysis buffer (150 mM NaCl, 50 mM Tris, 1% Triton^®^ X-100, 10 μM MG132, 100 mM NEM, pH 8.0) and pre-cleared by centrifugation at >14,000*g* for 15 min at 4°C. The protein concentration in each cleared supernatant was quantified using the BCA™ protein assay kit. Each sample was diluted in additional lysis buffer to adjust each sample so that it has an equal concentration of protein in 1 ml of total lysis buffer (typically 1 mg/ml). Forty microlitre of *myc*-coated magnetic beads (μMACS myc Kit; Miltenyi Biotec) were added to the supernatants, incubated 1 h at 4°C with rotation and loaded onto μMACS columns (Miltenyi Biotec). Immunoprecipitates were washed twice and eluted in loading buffer according to the manufacturer’s instructions prior to SDS-PAGE and western blotting with pp65 and HA antibodies.

### RNA interference

RNA interference (RNAi) oligonucleotides specific for PA28-α (L-012254-00), PA28-β (L-011370-01), LMP2 (L-006023-00), MECL1 (L-006019-00), LMP7 (L-006022-00), Rpn10 (L-011365-00) and NUB1/NUB1L (L-019158-00) were all purchased from Dharmacon. Non-targeting control siRNA (D-001810-10) were also used in each experiment and also obtained from Dharmacon. Briefly, HeLa A2+ and/or HEK293 cells were seeded in six-well plates and transiently transfected with non-targeting or targeting siRNA at a final concentration of 100 nM by using the Xtremgene kit (Roche), according to the manufacturer’s protocol. The knockdown of the specified protein was determined by western blotting using the appropriate antibody. For DC transfection, 4 × 10^7^ cells were resuspended in 100 μl Opti-MEM without red phenol (Invitrogen) and transferred into a 4-mm electroporation cuvette (Biorad) with 1,000 nmol siRNA duplex. The electroporator (Genepulser; Biorad) used a square-wave pulse of 500 V for 1 ms. Cells were then immediately transferred into 4 ml of RPMI 1640 with 10% FCS, containing GM-SCF and IL-4.

### Antigen presentation assay

HeLa A2+ or HeLa A2+/IP were transiently transfected with pp65, FAT10-pp65 and Ub-pp65 and used as targets cells for their potential to activate the production of IFN-γ by the pp65 CD8+ T cell clone 61. Following 4 h of transfection, target cells were serially diluted and then co-cultured with a fixed amount of T cells, resulting in graded effector-to-target (E:T) ratio in a final volume of 100 μl of RPMI 1640 supplemented with 10% FCS on U-bottom 96-well plates. After 16 h of incubation, the supernatants were collected and the IFN-γ content was determined using a commercially available human ELISA kit according to the manufacturer’s instructions. The data in the figures refer to the mean of two replicates. The SD was below 5% of the mean.

### Cross-presentation assay

Whole cell lysates were used as pp65 antigen sources for cross-presentation and prepared by four cycles of rapid freeze/thaw lysis of HeLa cells transiently transfected with pp65, Ub-pp65 or FAT10-pp65. Immature DC from HLA-A2+ donors were plated in duplicate on a 96-well plate at 50,000 cells per well and incubated for various periods of time with the various whole cell lysates in the presence of the pp65 CD8+ T cell clone 61 at different responder-to-stimulator ratio in a final volume of 200 μl. Alternatively, DC were used as unloaded or after being pulsed with 1 μM of the pp65_495–503_ synthetic peptide NLVPMVATV in the presence of LPS (1 μg/ml).

### Statistical analysis

Student’s *t* test (one-tailed) was used for data analysis when appropriate.

## Results

### FAT10 modification of pp65 improves the presentation of the HCMV pp65_495–503_ epitope

**Fig. 1:**
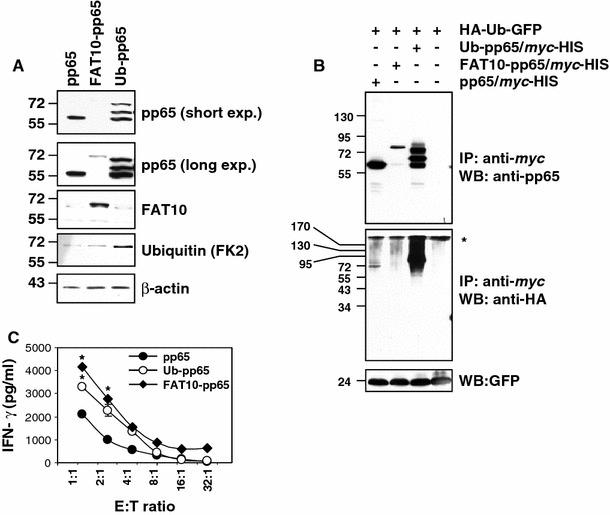
Increased reactivity of the pp65_495–503_-specific CTL clone 61 by HeLa A2+ cells transiently expressing either Ub-pp65 or FAT10-pp65. **a** HeLa A2+ cells were transfected with the various plasmids for 24 h and whole cell extracts were mixed with sample buffer, followed by SDS-PAGE separation and western blotting with* pp65*,* Ubiquitin * (*FK2*) and* FAT10* antibodies. Loading control was ensured by probing the membrane with the anti-β-actin mAb. **b** HeLa cells were transfected with* HA-Ub-GFP* alone or in combination with* pp65-* *myc*,* Ub-pp65-* *myc* or* FAT10-pp65-* *myc* for 16 h after which they were subjected to a 6-h treatment with 10 μM MG-132. Following incubation, cells were harvested and subjected to immunoprecipitation with *myc*-coated magnetic beads and western blot analysis with anti-HA (against Ub) and anti-pp65 mAb, as indicated. Loading control in this experiment was ensured by monitoring the expression levels of the GFP protein, which is immediately cleaved from the HA-Ub-GFP fusion product by hydrolases shortly after synthesis. **c** Following a 4-h transfection, HeLa A2+ cells expressing pp65, Ub-pp65 or FAT10-pp65 were washed and added to the pp65 CTL clone 61 recognising the HLA-A2-restricted pp65_495–503_ epitope at various E:T ratio for 16 h. The activation of the CTL clone 61 was assessed by measuring IFNγ content in the supernatant by ELISA (*p < 0.01, compared with untagged pp65, *n* = 3)

Because both Ub and FAT10 serve as signals for proteasome-dependent degradation, we compared their impact on MHC class I presentation. To this end, fusion proteins consisting of the HCMV-derived pp65 antigen N-terminally tagged with either Ub or FAT10 were expressed in HeLa A2+ cells. Monitoring the steady-state levels of the different pp65 constructs revealed that the expression level of FAT10-pp65 was strongly reduced when compared to that of the untagged pp65 (Fig. 1a). Importantly, the transcriptional activity of these two plasmids was identical (Fig. S1A). Furthermore, the expression level of pp65 and FAT10-pp65 in the detergent-insoluble fraction showed no significant differences (Fig. S1B), indicating that the cellular distribution of both of these constructs was similar. Taken together, these data strongly suggest that reduced signal for the FAT10-pp65 fusion protein observed in the detergent-soluble fraction reflects higher protein turnover. Detection of the Ub-pp65 fusion protein revealed the expected efficient initiation of poly-Ub chain formation in vivo, as shown by a typical 8-kDa ladder of high molecular weight bands detected with the pp65 antibody. Of the three bands detected, the lower one had the same molecular size as the untagged pp65, indicating that the Ub is partially removed by de-ubiquitylating enzymes (DUB). Of note, double conversion of glycine 75 and 76 to alanine and valine at the isopeptidase site (UbAV-pp65) did not efficiently block the Ub cleavage from the fusion protein, as determined by western blotting (Fig. S2A).

To analyze the ubiquitylation state of our different pp65 constructs, HeLa cells were transfected with HA-Ub-GFP alone or in combination with pp65-*myc*, Ub-pp65-*myc* or FAT10-pp65-*myc* for 16 h and were subsequently subjected to a 6-h treatment with 10 μM MG-132. Following incubation, the pp65 constructs were immunoprecipitated using *myc* magnetic beads and analysed by immunoblotting with anti-HA (against Ub) and anti-pp65 antibodies. As shown in Fig. 1b, N-terminal tagging of pp65 with Ub results in a strong poly-ubiquitylation of the Ub-pp65 fusion protein. A prolonged exposure of the western blot with the anti-pp65 antibody reveals that at least four Ub moieties are attached to the Ub-pp65 construct in these cells (Fig. S3). In contrast, the untagged pp65 and the FAT10-pp65 constructs were only slightly and similarly ubiquitylated.

To test the impact of pp65, Ub-pp65 and FAT10-pp65 on the presentation of the pp65_495–503_ epitope in HeLa A2+ cells, pp65 epitope presentation was monitored using a CD8+ T cell clone (CTL clone 61) that specifically recognises the immunodominant pp65_495–503_ epitope. To prevent saturation levels of MHC class I/peptide complexes, HeLa A2+ cells were transfected with each construct for only 4 h. As shown in Fig. 1c, in comparison to the untagged pp65, the FAT10-pp65 fusion protein enhanced antigen presentation approximately twofold and activated the CTL clone 61 to a similar extent as that seen with the Ub-pp65 fusion protein. Importantly, the improved pp65_495–503_ presentation obtained with either Ub-pp65 or UbAV-pp65 was substantially reduced when all the seven lysine residues of the fused Ub moiety were changed into arginine residues (UbK0-pp65) (Fig. S2B and S2C). These data formally show that the enhanced pp65 CTL response initiated by Ub-pp65 relies on the poly-ubiquitylation of its N-terminal Ub.

### The pp65_495–509_ epitope presentation derived from FAT10-pp65 is less dependent on Rpn10 than that derived from Ub-pp65

The observation that the FAT10- and Ub-pp65 fusion proteins similarly supported pp65_495–503_ epitope presentation raised the question concerning a putative receptor for 26S proteasome targeting. A major 26S proteasome receptor for poly-ubiquitylated substrates is the Rpn10 subunit of the 19S regulatory particle, originally called S5a [25, 26].

**Fig. 2:**
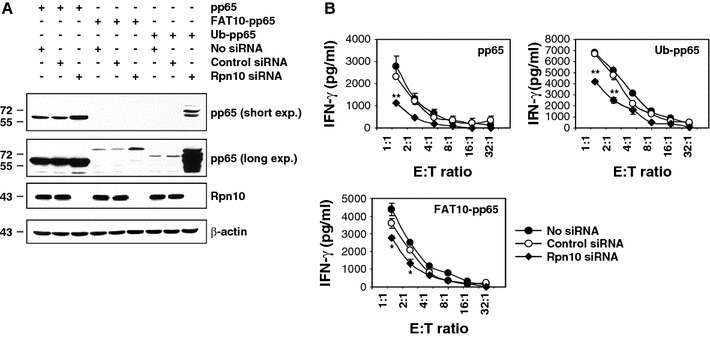
Rpn10 knockdown in HeLa A2+ cells leads to impaired presentation of the pp65_495–503_ epitope generated from pp65, Ub-pp65 or FAT10-pp65 proteins. **a** The role of Rpn10 in the turn-over of the* pp65*,* Ub-pp65* or* FAT10-pp65* substrates was examined by treating HeLa A2+ cells with either* control* or* Rpn10 siRNA* for 4 days followed by a subsequent transfection with the pp65, Ub-pp65 or FAT10-pp65 plasmids for 24 h, as indicated. The gene silencing efficiency for Rpn10 was determined by western blotting using a monoclonal antibody against Rpn10. The influence of Rpn10 depletion on the steady-state level of pp65, Ub-pp65 and FAT10-pp65 was examined by western blotting using the anti-pp65 antibody. **b** Rpn10-depleted HeLa A2+ cells were transfected with the pp65, Ub-pp65 or FAT10-pp65 plasmids for 4 h and used as target cells in a 16-h co-culture against the CTL clone 61. Controls in this CTL assay consist in the use of HeLa A2+ cells expressing pp65, Ub-pp65 or FAT10-pp65 which have been treated with non-targeting siRNA (*control siRNA*) or left untreated (*no siRNA*). The activation of the CTL clone 61 was assessed by measuring the IFN-γ content in the supernatant by ELISA. (*p < 0.01 and **p < 0.005, compared to untreated cells or cells treated with control siRNA, *n* = 2)

To determine whether Rpn10 may also serve as a receptor for FAT10-modified substrates, we determined the steady-state level of pp65, Ub-pp65 and FAT10-pp65 in Rpn10-siRNA-silenced HeLa A2+ cells. As illustrated in Fig. 2a, RNAi treatment against Rpn10 for 96 h resulted in an almost complete depletion of the Rpn10 subunit. The Rpn10 knockdown was associated with a substantial stabilisation of Ub-pp65 and FAT10-pp65 indicating that both of these fusion proteins are targeted to Rpn10 for proteasomal degradation. The steady-state level of the untagged pp65 in Rpn10-depeleted cells was also significantly affected which is in line with the observation that wild-type pp65 undergoes poly-ubiquitylation in vivo. Nevertheless, the stabilisation of FAT10-pp65 observed in the absence of Rpn10 was less pronounced than that observed for the two other pp65 constructs, suggesting a reduced affinity of FAT10-pp65 for Rpn10 or that Rpn10 is not the only FAT10 interacting 26S proteasome subunit.

Next, we tested whether Rpn10 silencing may exert any effect on the presentation of the pp65_495–503_ epitope arising from our various pp65 constructs. As shown in Fig. 2b, Rpn10 down-regulation resulted in a significant impairment of pp65_495–503_ epitope presentation deriving from the untagged pp65 protein and the Ub-pp65 fusion protein. Impairment of Rpn10 expression also affected the efficiency of pp65 epitope presentation exerted by the FAT10-pp65 fusion protein. However, the observed decrease of pp65 epitope presentation was less pronounced, which appears to be in concordance with the reduced stabilization of FAT10-pp65 upon Rpn10 deficiency demonstrated in Fig. 2a. These data not only reveal that Rpn10 is involved in the proteasomal degradation of pp65 and Ub-pp65 but also suggest that Rpn10 can act as receptor for FAT10-modified substrates.

### FAT10-pp65 processing is not controlled by i-proteasomes and/or PA28

**Fig. 3:**
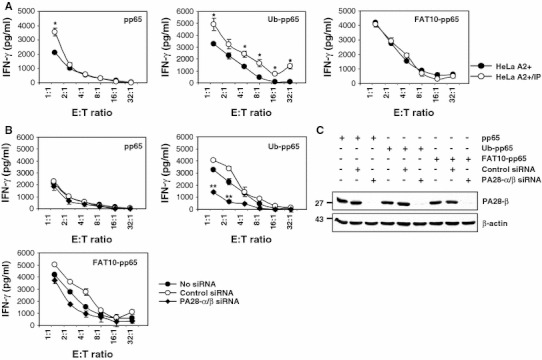
The presentation of the pp65_495–503_ antigenic peptide arising from the FAT10-pp65 fusion protein is not influenced by i-proteasomes and/or PA28. **a** The role of i-proteasomes in the presentation of the pp65_495–503_ antigenic peptide arising from the* pp65*,* Ub-pp65* and* FAT10-pp65* fusion proteins was assessed in a 16-h CTL assay using targets cells consisting of HeLa A2+ cells having dominantly s-proteasomes or HeLa A2+/IP cells over-expressing the three inducible subunits LMP2, MECL1 and LMP7, as indicated (*p < 0.01, compared with HeLa A2+ having predominantly a s-proteasome, *n* = 3). **b** HeLa A2+ cells were left untreated (*no siRNA*) or treated with non-targeting siRNA (*control siRNA*) or a combination of siRNA specific for PA28-α and PA28-β. After 48 h, these cells were subjected to a short 4-h transfection with the* pp65*,* Ub-pp65* and* FAT10-pp65* plasmids and used as target cells in a CTL assay for monitoring the presentation of the pp65_495–503_ peptide, as described in **a** (**p < 0.005, compared with untreated cells or cells treated with control siRNA, *n* = 3). **c** The content of HeLa A2+ for PA28 was analysed by western blotting using an antibody specific for* PA28-β*. Anti-β-actin antibody was used to ensure equal protein loading

In many cases, i-proteasomes or the proteasome activator PA28 positively influence presentation of epitopes arising from poly-ubiquitylated antigens [27, 28]. Therefore, we next studied the role of i-proteasomes in the processing of FAT10-pp65 in HeLa A2+/IP cells that predominantly express i-proteasomes [24]. As shown in Fig. 3a, expression of either pp65 or Ub-pp65 in HeLa A2+/IP resulted in improved antigen presentation of the pp65_495–503_ epitope, when compared to HeLa A2+ control target cells that predominantly express s-proteasomes. In striking contrast, the presence of i-proteasomes exerted no effect on the efficiency of the FAT10-mediated pp65 epitope presentation, indicating that the molecular prerequisites for FAT10-dependent antigen processing differ from those of the Ub-dependent pathway.

**Fig. 4:**
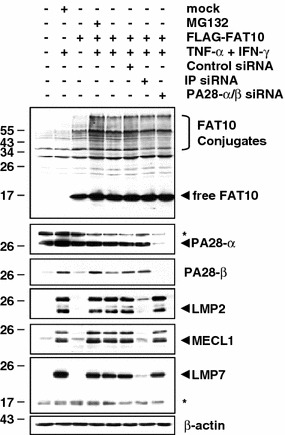
The expression pattern of FAT10-protein conjugates in response to TNF-α and IFN-γ is not altered by depletion of either i-proteasomes or PA28. The role of PA28 and the i-proteasome in the steady-state level of the accumulated FAT10-modified substrates was assessed by treating HEK293 cells with siRNA specific for PA28-α/β or for the three inducible proteasomal subunits LMP2/MECL1/LMP7 (IP), respectively. After 24 h, cells were treated with a combined treatment of TNF-α and IFN-γ or left untreated prior to a subsequent 24-h transfection with the flag-tagged FAT10 plasmid. The membrane shown was subjected to western blot analysis using antibodies against* FAT10*,* PA28-α*,* PA28-β*,* LMP2*,* MECL1* and* LMP7*, as indicated. Equal loading was determined by using anti-β-actin mAb

We next studied the effects of PA28 on the presentation of the pp65_495–503_ epitope derived from the FAT10-pp65 fusion protein. To deprive the target cells of PA28, both PA28-α and PA28-β were silenced by siRNA. Cells transfected without siRNA or non-targeting siRNA oligonucleotides (control siRNA) were used as controls. Following successful down-regulation of PA28 (Fig. 3c), HeLa A2+ cells were transfected with pp65, Ub-pp65 or FAT10-pp65 and the pp65_495–503_ CTL response was monitored by IFN-γ release assays. As shown in Fig. 3b, the presentation of the pp65_495–503_ epitope liberated from untagged pp65 or the FAT10-pp65 fusion remained largely unaffected in PA28-depeleted HeLa A2+ cells. In contrast, the presentation of the pp65_495–503_ epitope derived from the Ub-pp65 fusion protein was considerably reduced following down-regulation of PA28 expression in HeLa A2+ cells. Taken together, these data demonstrate that neither i-proteasomes nor PA28 significantly influence the processing of FAT10-modified substrates for MHC class I presentation. This conclusion finds further support by the observation that the accumulation of FAT10-modified proteins following a combined treatment of TNF-α and IFN-γ is not altered in i-proteasome- or PA28-depleted HEK293 cells (Fig. 4).

### NUB1 and its splicing variant NUB1L specifically regulate the pp65_495–503_ presentation arising from FAT10-pp65

**Fig. 5:**
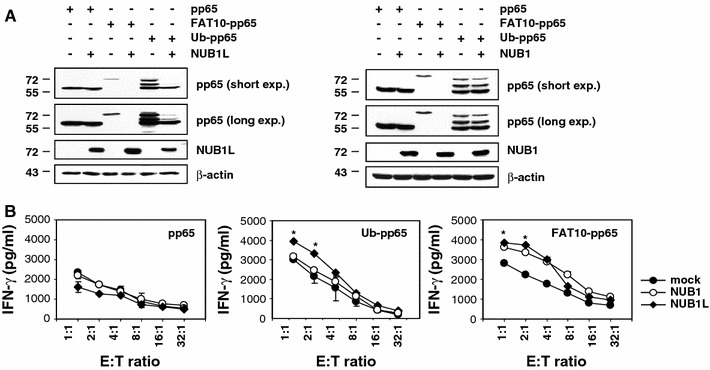
NUB1 and NUB1L accelerate the protein turnover of FAT10-pp65 which results in improved presentation of the pp65_495–509_ antigenic peptide in HeLa A2+ cells. **a** Untagged versions of* NUB1* and* NUB1L* were co-expressed together with* pp65*,* Ub-pp65* or* FAT10-pp65* in HeLa A2+ cells and their respective influence on the steady-state level of each pp65 antigenic variant was determined by western-blot using an anti-pp65 monoclonal antibody. The over-expression of NUB1 and NUB1L was checked by probing the membrane with a polyclonal antibody recognising both NUB1 and NUB1L (equal protein loading was ensured by using the anti-β-actin mAb). **b** The effects of NUB1 and NUB1L over-expression were also tested for the presentation of the pp65_495–503_ peptide emerging from* pp65*,* Ub-pp65* or* FAT10-pp65*. HeLa A2+ cells were transfected with either the pcDNA3.1 empty vector (*mock*),* NUB1* or* NUB1L* for 24 h prior to a subsequent transient transfection with the pp65, Ub-pp65 or FAT10-pp65 constructs for 4 h. Cells co-expressing NUB1 or NUB1L and the various pp65 antigenic forms were subsequently tested for their capacity to present the pp65_495–503_ antigenic peptide using the CTL clone 61 in a 16-h IFN-γ release assay (*p < 0.01, compared to HeLa A2+ cells transfected with the pcDNA3.1 empty vector, *n* = 2)

Both the Ub-binding proteins NUB1 and its splicing variant NUB1L have been shown to accelerate the degradation of FAT10 [29] and thus appear to be important regulators of both poly-Ub- and FAT10-dependent protein degradation. We therefore next investigated the effects of NUB1 and NUB1L on the steady-state levels of pp65, Ub-pp65 and FAT10-pp65. Untagged versions of NUB1 or NUB1L were co-expressed in HeLa A2+ cells together with pp65, Ub-pp65 or FAT10-pp65 and their relative expression levels were monitored by western blotting using a monoclonal anti-pp65 antibody. In accordance with previous studies [30], both NUB1 and NUB1L significantly enhanced the degradation rate of the FAT10-pp65 fusion protein (Fig. 5a). However, only NUB1L significantly accelerated the turnover of the Ub-pp65 fusion protein, suggesting that NUB1 and NUB1L differ in their ability to shuttle Ub substrates for proteasomal degradation. Coincidently, over-expression of either NUB1 or NUB1L resulted in substantially improved presentation of the FAT10 derived pp65_495–503_ antigenic peptide (Fig. 5b). Likewise, over-expression of NUB1L together with Ub-pp65 was accompanied by an increased activation of the pp65_495–503_ CD8+ T-cell clone. By contrast, the over-expression of either NUB1 or NUB1L had no substantial effect on the pp65_495–503_ antigen presentation arising from the untagged pp65 (Fig. 5b).

**Fig. 6:**
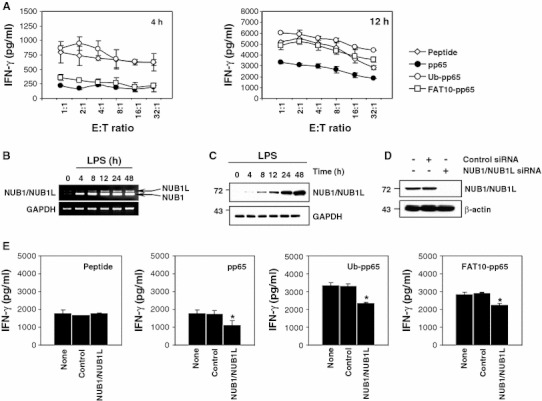
Both FAT10 and Ub facilitate cross-presentation of the pp65_495–503_ epitope by DC when fused to the N-terminus of the HCMV-derived pp65 protein. **a** Immature HLA-A2+ DC were fed with* pp65*,* Ub-pp65* or* FAT10-pp65* necrotic HeLa cells at a ratio at 2 per DC and simultaneously exposed to LPS (1 μg/ml) to induce maturation. The ability of pp65-, Ub-pp65- and FAT10-pp65-pulsed DC to stimulate the CTL clone 61 was tested in a 4- or 12-h IFN-γ release assay, as indicated. Internal controls consist in DC loaded with 1 μm of the 9-mer synthetic pp65_495–503_ peptide (*p < 0.01, compared to DC loaded with untagged pp65, *n* = 2). **b** RT-PCR analysis of NUB1 and NUB1L transcripts in LPS-treated DC following various periods of time. The amplified RT-PCR products NUB1 and NUB1L were separated on 2% agarose gels and visualised by ethidium bromide. Electrophoresis of the PCR products showed* NUB1* (222 bp) band and a larger band corresponding to* NUB1L* (256 bp). PCR using* GAPDH* showed similar amplification levels among the samples tested. **c** Time-course induction of NUB1 and NUB1L in DC following LPS stimulation as assessed by western-blotting. Day 5-immature DC were treated with 1 μg/ml LPS for various periods of time, as indicated. Thirty micrograms of total cellular proteins were loaded in each lane and resolved 12% SDS-PAGE gel followed by western blotting with a polyclonal antibody specific for both* NUB1* and* NUB1L*. Equal amounts of proteins was ensured using* GAPDH* antibody. **d** Western blot analysis for NUB1/NUB1L in untreated DC or DC treated with* control siRNA* or* NUB1/NUB1L*-specific* siRNA*, as indicated. **e** HLA-A2+ DC with a knockdown of NUB1/NUB1L were loaded with 1 μM of the pp65_495–503_ synthetic* peptide* or cell-associated* pp65*,* Ub-pp65* and* FAT10-pp65* and subsequently cultured with the CTL clone 61 at a E:T ratio of 1:1 in the presence of LPS (1 μg/ml). Cross-presentation of the pp65_495–503_ peptide was assessed by measuring the IFN-γ content in the supernatant following 12 h of co-culture (*p < 0.01, compared to DC treated with control siRNA, *n* = 2)

To test and compare the impact of pp65, Ub-pp65 and FAT10-pp65 on cross-presentation, monocyte-derived DC were loaded with whole cell lysates containing pp65, Ub-pp65 or FAT10-pp65, co-cultured with the pp65 CTL clone 61 in the presence of LPS, and IFN-γ release was assayed, as previously described [14, 31]. As shown in Fig. 6a, DC fed with either FAT10-pp65 or Ub-pp65 proteins elicited significant responses following 12 h of co-culture which almost equalled those measured with the synthetic 9-mer pp65 peptide, but which were nearly twofold greater than those observed with the untagged pp65 protein. Interestingly, the enhancement of pp65 cross-presentation elicited by the Ub-pp65 was already visible after 4 h, while enhancement of the immune response elicited by the FAT10-pp65 fusion protein became detectable after 12 h of co-culture. Importantly, the early pp65 CTL response observed with Ub-pp65 was abrogated when using prefixed DC (Fig. S4), indicating that the pp65 cross-presentation initiated in the first 4 h of co-culture requires processing and is not due to peptide transfer from the antigen-donor cells to the DC cell surface HLA-A2+ molecules.

Because of the observation that both NUB1 and NUB1L accelerate the degradation of FAT10-pp65, we next aimed to estimate the transcription level of both of these proteins in maturing DC following LPS stimulation using RT-PCR. As shown in Fig. 6b, NUB1 and, to a much lesser extent, NUB1L were induced in LPS-treated DC from 4 h of stimulation. However, we were unable to determine whether both abundant splice variants of the two NUB1 isoforms were translated because they were indistinguishable in western blot analyses (Fig. 6c). To address the role of the increased expression of NUB1 and NUB1L in cross-presentation, day-5 immature DC were electoporated with siRNA specific for NUB1/NUB1L for 24 h prior to a subsequent stimulation with LPS, which resulted in a strong impairment of NUB1/NUB1L up-regulation (Fig. 6d). Strikingly, the pp65_495–503_ cross-presentation arising from DC fed with pp65, Ub-pp65 or FAT10-pp65 was reduced by about 20% in NUB1/NUB1L-depleted DC (Fig. 6e), demonstrating the contribution of NUB1/NUB1L to proteasome-dependent cross-presentation of the pp65_495–503_ epitope.

## Discussion

FAT10 is the only UBL so far known that shares with Ub the capacity of targeting substrates for proteasomal breakdown. However, very little is known about its function and nothing is known about its ability to support the generation of peptides suitable for MHC class I presentation. Here, we demonstrate that FAT10 modification of the HCMV-derived antigen pp65 (FAT10-pp65) enhances the presentation of the HLA-A2-restricted pp65_495–509_ antigenic peptide (Fig. 1c) and provide evidence that FAT10-pp65 differs from Ub-modified pp65 in using the proteasome machinery. The pp65_495–503_ presentation obtained with Ub-pp65 is improved by approximately 50%. This is less than originally observed by Townsend and colleagues with the NP_365–370_ peptide using a Ub-Arg-NP fusion protein (N-end rule) [1]. However, this discrepancy can be explained by the fact that, in contrast to the pp65_495–503_ presentation, the NP_365–370_ presentation is defective and, as such, increases much more dramatically following N-terminal Ub fusion.

While this paper was under revision, an interesting study of Buchsbaum and co-workers reported that the increased degradation rate of a FAT10-GFP fusion protein is facilitated by poly-ubiquitylation [32]. Our data show that our FAT10-pp65 fusion protein is not more poly-ubiquitylated than the untagged pp65 (Fig. 1c; Fig. S3), suggesting that the accelerated degradation of this construct cannot be attributed to its enhanced poly-ubiquitylation state. Our results do not formally exclude a possible involvement of the Ub-conjugation system in the regulation of the breakdown of FAT10-pp65. Nevertheless, our results would still imply that the processing of a substrate bearing simultaneously FAT10 and Ub differs from that of Ub-tagged or posttranslationally Ub-modified proteins.

Also, the FAT10-pp65 fusion protein expressed in HeLa cells improved cross-presentation of the pp65 epitope by LPS-stimulated DC demonstrating that FAT10 modification provides an alternative signal for efficient antigen processing and subsequent MHC class I presentation.

Induction of FAT10 synthesis requires IFN-γ and TNF-α [22], which also trigger the synthesis of i-proteasomes and the proteasome activator PA28. Despite this, and in striking contrast to poly-ubiquitylated pp65, the FAT10-dependent pp65 epitope presentation was already most efficient in the presence of s-proteasomes and was not further enhanced by i-proteasomes or PA28 (Fig. 3a, b). These data suggest that the degradation of the FAT10- and Ub-protein conjugates are governed by different molecular mechanisms. One possible explanation for this surprising result may be that FAT-10- and Ub-modified proteins interact with the 26S proteasome in different ways. Interestingly, our experiments show that both FAT10-pp65 and Ub-pp65 share the 19S regulator subunit Rpn10 (Fig. 2a), known to bind poly-Ub-chains as interaction partner [25, 26]. However, siRNA experiments also revealed that, while Rpn10 deficiency exerts profound negative effects on the presentation of the pp65 epitope derived from Ub-pp65, the effect on the FAT10-pp65-derived epitope is considerably less pronounced (Fig. 2b). In light of the extremely efficient proteasome-dependent turnover of FAT10-pp65, this may indicate that FAT10 binds Rpn10 less efficiently and/or that Rpn10 is not the only and not the decisive interaction partner of FAT10 modified proteins within the 26S proteasome complex.

So far, NUB1L and its natural splicing variant NUB1 (which has a deletion of 14 amino acids) had been the only proteins identified to ferry FAT10 for proteasome-dependent degradation [22, 30]. Our data further support a role for both NUB1 and NUB1L in facilitating the breakdown of FAT10-modified substrates, as evidenced by decreased steady-state levels of the FAT10-pp65 fusion protein in cells over-expressing NUB1 or NUB1L (Fig. 5a). Importantly, the accelerated degradation of FAT10-pp65 by NUB1 or NUB1L was accompanied by a marked increase of the pp65_495–503_ CTL response (Fig. 5b). However, our experiments rule out an entirely overlapping function of NUB1 and NUB1L because only NUB1L was found to enhance the turnover of the Ub-pp65 fusion (Fig. 5a). Thus, unlike NUB1 (which appears FAT10-specific), NUB1L seems to be positioned at the intersection of the Ub and FAT10 pathways, suggesting a model in which the accelerated disposal of Ub-modified proteins by NUB1L is connected to an increased supply of antigenic peptides for MHC class I presentation.

The optimal form of the antigenic source for effective cross-priming is still a matter of debate, ranging from stable antigens as being a favourable source for cross-presentation [33–35] to unstable proteins, including defective ribosomal products (DriPs), being more effective than mature proteins for stimulating cross-priming [36, 37]. Our data show that both Ub and FAT10 fusion proteins serving as vehicles for pp65 delivery into DC were by far superior in activating CTL to the untagged pp65 (Fig. 6a). This data would support the notion that short-lived proteins are better cross-presented than the stable ones. Interestingly, there exist kinetic differences in enhancing cross-presentation, with cross-presentation from the FAT10-pp65 fusion being considerably lower during the first 4 h of the assay than that observed with Ub-pp65. However, Ub-pp65 and FAT10-pp65 exerted almost identical cross-presentation efficiency after 12 h of co-culture.

Interestingly, NUB1 and, to a lesser extent, NUB1L are induced during the course of LPS-induced DC maturation. The fact that at the transcriptional level the NUB1/NUB1L ratio was close to 10 (Fig. 6b, c), seems to suggest that NUB1 is the major isoform in mature DC silencing of NUB1/NUB1L expression and was accompanied by a slight but significant decrease of the pp65 cross-presentation levels by DC (Fig. 6e). Surprisingly, however, impairment was not restricted to DC loaded with FAT10-pp65 but was also observed to a similar extent with DC loaded with either untagged pp65 or Ub-pp65. This appears to contrast with the observation that NUB1 and/or NUB1L can only enhance MHC class I presentation of FAT10 substrates. However, given the sensitivity of the assay, the low amounts of NUB1L expressed during DC maturation are probably sufficient to influence processing of the pp65 epitope derived from untagged pp65 and Ub-pp65. Alternatively, the sensibility of pp65 and Ub-pp65 to NUB1/NUB1L down-regulation may indicate that both forms of the pp65 antigen undergo FAT10 modification within DC prior to proteasomal degradation for cross-presentation. Proteasome-dependent cross-presentation was previously estimated to only contribute to total cross-presentation to approximately 30% [38]. Keeping this in mind, the observed inhibitory effect on cross-presentation of about 20% exerted through the knockdown of NUB1/NUB1L support the central role of the two proteasomal adapter proteins in antigen cross-presentation.

Collectively, our findings outline the FAT10-NUB1/NUB1L-Rpn10 axis as a novel route for MHC class I antigen direct presentation and DC-based cross-presentation. Considering the strongly delayed expression of FAT10 upon cytokine stimulation, the existence of this new route may allow presentation of antigenic peptides that would not be generated in adequate amounts by the classical Ub-conjugation pathway. Nevertheless, the contribution of each pathway to antigen presentation is difficult to assess. The moderate or absent phenotype exhibited by FAT10-deficient mice [19] suggests that the FAT10-conjugation pathway may not be a privileged and/or the predominantly used pathway for MHC class I presentation. This hypothesis is further supported by the observation that FAT10 is not expressed in cells under normal conditions. It is instead conceivable that the FAT10-conjugation machinery represents a complementary pathway which is used when Ub availability becomes a rate-limiting factor. Interestingly, FAT10 is up-regulated at the transcriptional level by cytokines (i.e., TNF-α and/or IFN-γ), which are also thought to deplete the pool of free Ub by increasing the formation of Ub-protein conjugates in immune cells [14, 39] as well as in non-immune cells [24]. Therefore, the role of the FAT10-dependent degradation machinery may be to support an overloaded Ub pathway in the removal of damaged proteins in later phases of stimulation. For the same reason, this route may be up-regulated in some pathological conditions such as viral infections and/or tumours, and may explain the observation that FAT10 is over-expressed in tumours exhibiting an alteration of the Ub-conjugation system such as in gastric cancer [17, 40]. Taken together, these data highlight the FAT10-dependent degradation machinery as a distinct MHC class I antigen processing pathway and suggest new avenues for FAT10-based immunotherapy in viral infections as well as in anti-tumour vaccinations.

## Electronic supplementary material

Below is the link to the electronic supplementary material.

Supplementary material 1 (DOC 886 kb)

## Abbreviations

DUB: De-ubiquitylating enzyme
FAT10: HLA-F adjacent transcript 10
NUB1: NEDD8 ultimate buster 1
PA28: Proteasome activator 28
pp65: Phosphoprotein 65
Ub: Ubiquitin
UBL: Ubiquitin-like modifier
UPS: Ubiquitin–proteasome system
