# Supplementary material for: The FAT10- and ubiquitin-dependent degradation machineries exhibit common and distinct requirements for MHC class I antigen presentation
Source: Cell Mol Life Sci. 2012 Feb 19;69(14):2443–54. doi: 10.1007/s00018-012-0933-5 (PMC3383951; doi:10.1007/s00018-012-0933-5)
Supplement: Supplementary file 1 — Supplementary material 1 (DOC 886 kb) [file 18_2012_933_MOESM1_ESM.doc]

**The FAT10- and ubiquitin-dependent degradation machineries exhibit common and distinct requirements for MHC class I antigen presentation.**

Frédéric Ebstein, Andrea Lehmann and Peter-Michael Kloetzel.

**Supplemental Material :**

**Fig S1**: **Modification of pp65 with either Ub or FAT10 does not affect its distribution in NP40–insoluble cellular compartments.** *A*. Semi-quantitative PCR analysis of pp65 mRNA levels in HeLa cells following a 24-h transfection with plasmids encoding pp65, Ub-pp65 or FAT10-pp65. GAPDH was used as an internal control to standardise mRNA quantity. PCR cycle number was 30 for both pp65 and GAPDH. *B*. Western-blot analysis of the insoluble fractions (pellet) from HeLa cells expressing pp65, Ub-pp65 or FAT10-pp65. For preparation of detergent-insoluble cell extracts, cells were lysed with 0.1% NP40 and soluble proteins were recovered as supernatants following centrifugation for 15 min at 14.000 rpm. Lysis buffer supplemented with 4 % SDS was added to the remaining pellet to give the insoluble fraction. Immunoblot analysis was performed using the monoclonal anti-pp65 antibody as well as the anti-actin antibody to ensure equal protein loading.

**Fig S2**: **The improved pp65(495-503) presentation emerging from the Ub-pp65 construct requires cellular poly-ubiquitylation of the fused Ub moiety.** *A*. HeLa A2+ cells were transfected with Ub-pp65 or a non-cleavable UbAV-pp65 mutant for 24 h and whole cell extracts were mixed with sample buffer, followed by SDS-PAGE separation and western blotting with pp65. The UbAV-pp65 fusion protein was constructed with pp65 fused to Ub with a double conversion of the GG^75, 76^ at the isopeptidase site by AV. Loading control was ensured by probing the membrane with the anti-β-actin mAb. *B*. The expression pattern of Ub-pp65 or a UbK0-pp65 mutant devoid of lysine residues was analysed in HeLa A2+ cells by western-blotting using an anti-pp65 monoclonal antibody. The UbK0-pp65 construct was obtained by substituting all 7 lysines residues of the fused Ub moiety into arginine residues. *C*. Following a 4-hr transfection, HeLa A2+ cells expressing pp65, Ub-pp65, UbAV-pp65 or UbK0-pp65 were washed and added to the pp65 CTL clone 61 recognising the HLA-A2-restricted pp65_495-503_ epitope at various E:T ratio for 16 h. The activation of the CTL clone 61 was assessed by measuring IFN−γ content in the supernatant by ELISA (**p*<0.01, compared with untagged pp65, *n*=2).

**Fig S3**: **The Ub-pp65 fusion protein bears at least 4 ubiquitin moieties in HeLa cells.** *Myc* (pp65) pull down as previously described in Fig. 1B. HeLa cells were transfected with HA-Ub-GFP alone or in combination with pp65-*myc*, Ub-pp65-*myc* or FAT10-pp65-*myc* for 16 hr before being subjected to immunoprecipitation with *myc*-coated magnetic beads and western-blot analysis (prolonged exposure) with anti-HA (against Ub) and anti-pp65 mAb, as indicated. Loading control in this experiment was ensured by monitoring the expression levels of the GFP protein.

**Fig S4**: **The increased pp65 cross-presentation capacity of DC loaded with Ub-pp65 after a 4-hr assay requires antigen processing.** To distinguish the pp65-endocytosed DC population versus the pp65 surface bound DC population, HLA-A2+ DC were prefixed for 1 min with 0,001% glutaraldehyde. After two washes with complete medium, immature prefixed or unfixed DC were fed with Ub-pp65 necrotic HeLa cells at a ratio at 2 per DC and simultaneously exposed to LPS (1 µg/ml) to induce maturation. The ability of Ub-pp65-pulsed DC to stimulate the CTL clone 61 was tested in a 4-hr IFN-γ release assay at the ratio 1:1. Internal controls consist in DC loaded with 1 µm of the 9-mer synthetic pp65_495-503_ peptide (***p*<0.005, compared to unfixed DC, *n*=2).
